# Guillain-Barre Syndrome Associated with Gastric Cancer: Paraneoplastic Syndrome or Immunological Disorder?

**DOI:** 10.4021/wjon259w

**Published:** 2011-01-01

**Authors:** Maria Colantuoni, Elide Matano, Salvatore Alfieri, Sabino De Placido, Chiara Carlomagno

**Affiliations:** a Department of Molecular and Clinical Endocrinology and Oncology, University Federico II, Naples, Italy

**Keywords:** Guillain Barre syndrome, Gastric cancer, Paraneoplastic syndrome

## Abstract

Guillain-Barre syndrome is a rare clinical entity classified as an ascending muscle paralysis led by autonomic nervous dysfunction due to autoimmune damage of peripheral nerves. Paraneoplastic Guillain-Barre syndrome has been described in association with some kinds of tumors (B-cell Lymphoma and small cell lung cancer). We describe the case of a 74-year-old woman affected by gastric adenocarcinoma, treated with surgery and adjuvant chemotherapy, who developed simultaneously skin cancer relapse and severe Guillain-Barre syndrome. Although the timing of clinical presentation suggests a paraneoplastic origin, other interesting features were present in this patient such as familial and personal anamnesis for autoimmune disease, HCV infection, and neurotoxic chemotherapy. According to literature, we investigated different pathogenetic hypothesis. According to the poorness of data, further investigations are necessary to establish a relationship between Guillain-Barre syndrome and gastric cancer.

## Introduction

Although incidence of gastric cancer is slowly decreasing over last decades, it is still a frequent tumor, with about 900.000 new cases/year in the world [1]. Unfortunately, most patients reach diagnosis with locally advanced or metastatic disease. Nodal involvement and blood diffusion are frequent and precocious events. Most common metastasis sites are lung, liver, peritoneum, and bone, less frequent sites are bone marrow, skin, central nervous system, and muscle. Paraneoplastic syndromes are quite rare in this kind of neoplasm. Nevertheless, gastric cancer has been associated with various paraneoplastic syndromes such as dermatomyositis, acanthosis nigricans, erythema giratum repens, trombophlebitis, Lambert-Eaton myasthenic syndrome, dementia, cerebellar ataxia, carcinoide syndrome, and ectopic adrenocorticotropic hormone syndrome. However, only few signalizations are available about Guillain-Barre syndrome [2].

## Case Report

A 74-year-old HCV-positive woman, affected by a not well defined psychiatric disorder well controlled with medical therapy (Risperidone 1 mg, 1 cp bid), in October 2008 underwent a total gastrectomy with histological diagnosis of adenocarcinoma, TNM stage IV (pT3, N2, G3, M1 due to the infiltration of a non regional node in the hepatoduodenal ligament). No residual disease or distant metastases were present at the postoperative total body CT scan.

From November 2008 to May 2009, the patient received 12 cycles of adjuvant chemotherapy with FOLFOX-4 (oxaliplatin, 5-fluorouracil, folinic acid) regimen. During the follow-up period, in July 2009, the patient suffered acute arthritis of the wrist and persistent diarrhea, thus she underwent colonoscopy plus biopsy and received the clinical and histological diagnosis of Enteroarthritis, treated with salazopirin, 1 cp bid.

In April 2010, the patient presented at our observation with skin nodules on the abdominal wall and neurological symptoms, including weakness and tremor starting in feet and legs and spreading to upper body and arms, hypotonia and areflexia of both legs and arms, ophthalmoplegia, lack of facial movement, difficulty in chewing and disphagia, and lack of bladder and anal control. All these clinical signs and symptoms suggested the diagnosis of ascending paralysis of Guillain-Barre. The total body CT scan did not show any metastatic localization of disease in liver, lung, or central nervous system. The biopsy of the skin nodules confirmed the histological diagnosis of cutaneous metastasis from gastric cancer. The analysis of cerebrospinal fluid collected during rachicentesis showed high levels of proteins and absence of lymphocytes (that is a typical scenario of Guillain-Barre syndrome). Viral antibodies titres for HSV 1, HSV 2, VZV, and CMV were negative both in serum and in cerebrospinal fluid. Serical levels of B12 vitamin and Folate were within normal range.

The patient soon started treatment with intravenous immunoglobulin (0.2 g/kg/die for 5 days) but the disease was very rapidly progressive and she died ten days after diagnosis for respiratory failure due to the paralysis of infracostal muscles.

## Discussion

Guillain-Barre disease is an acute inflammatory demyelinating polyneuropathy (AIDP), affecting the peripheral nervous system, usually triggered by an acute infectious process. Most common symptoms are weakness and loss of tendon reflexes starting in legs and spreading to upper limbs and face (ascending paralysis), lack of eyes and facial movement, disphagia, difficulty in chewing and loss of sphincter functions.

Diagnosis can be achieved by lumbar puncture (showing high rate of protein and no cells), electromyography and nerve conduction study. Although it is known that Guillain-Barre syndrome is an autoimmune disorder, the pathogenesis is probably multifactorial and still not well understood.

This syndrome has been described in patients with different types of neoplasm [3], especially non-Hodgkin lymphoma [4, 5], but few data are available about the real relationship between the tumor and the neurological disease, and many authors consider this association as casual. Different autoantibodies have been identified in patients affected by cancer and neuropathy, in particular, serum levels of anti-onconeural antibodies (anti-Hu, anti-CV2) seem to play a role in the genesis of the neurological disorder [6].

It’s known that lack of B12 vitamin, due to total gastrectomy, is able to cause severe neuropathy associated with anemia and/or glossitis. However, in our patient serum levels of B12 vitamin were normal; furthermore, the high albumin levels in the cerebrospinal fluid suggested an autoimmune pathogenesis rather than a lack of vitamins. In a Japanese study, seventeen patients with polineuropathy arisen after gastrectomy showed nearly normal concentration of all B-group vitamins except Thiamine (B1 vitamin) levels, which were significantly lower. All these patients had developed various degrees of motor and sensory impairment and some cases had progressed rapidly, mimicking Guillain-Barre syndrome. Substantial functional recovery was observed 3 to 6 months after initiating thiamine supplementation. These findings suggest that lack of B-vitamins other than cianocobalamine could play a role in neurological disorders associated with gastrectomy [7].

The dose-limiting toxicity of oxaliplatin is peripheral neuropathy. This adverse event can be acute and transient or dose-cumulative and persistent, as it can last for many months after the cessation of the drug administration [8-10]. To our knowledge, in the literature there is only one report of Guillain-Barre syndrome occurred to a patient receiving oxaliplatin for metastatic colon cancer. In this case, authors identified the increase of cytokine (such as TNF-α and IL-6) caused by oxaliplatin as a triggering agent for autoimmune reaction [11]. However, it is of note that, in this report, the patient was still receiving oxaliplatin when the neurological symptoms occurred, while in our patient, the neurological symptoms started about 1 year after oxaliplatin cessation.

Nevertheless, Nardone et al described four cases of acute sensorimotor neuropathy occurred several months after completion of an oxaliplatin based chemotherapy. In all these four patients, nerve conduction studies were performed. Sensory nerve conduction was abnormal in all patients, while in 2 patients, the electrodiagnostic studies showed a mixed axonal and demyelinating sensorimotor polyneuropathy [12].

Finally, other factors contributing to the aberrant immune response have to be considered as potential causes of the Guillain-Barre syndrome. Our patient had at least another autoimmune disease, the enteroarthitis. Collecting the familial anamnesis, we knew that one sister was affected by Multiple Sclerosis, which is a neurological autoimmune disorder. Genetic influence on autoimmune disease is well known. For instance, it is established the relationship between all these kind of disease and the HLA locus polymorphisms [13]. Some evidence are available about the association of HLA A3 and B8 with Guillain-Barre syndrome [14]. Moreover, in patients affected by autoimmune disease, a higher frequency of HCV infection can be found, compared with that of healthy controls. This difference is statistically significant (8.7 vs 0.4, p < 0.0001) for cryoglobulinemia, pemphigus vulgaris, secondary antyphospholipid syndrome, vasculitis, inflammatory bowel disease, and Hashimoto thyroiditis [15], but no data are available about Guillain-Barre syndrome.

In conclusion, the close cause-effect relationship between gastric cancer and Guillain-Barre syndrome remains to be demonstrated. This neurological disorder can be prompted by many effectors, including drugs affecting peripheral nerves. The most probable cause of the Guillain-Barre syndrome in patients with solid tumours can be the complex alteration of the immunologic system leading to the lack of control of the micrometastases and autoimmune manifestations.
